# Methodology for Single Bee and Bee Brain ^1^H-NMR Metabolomics

**DOI:** 10.3390/metabo11120864

**Published:** 2021-12-13

**Authors:** Jayne C. McDevitt, Riju A. Gupta, Sydney G. Dickinson, Phillip L. Martin, Jean Rieuthavorn, Amy Freund, Marie C. Pizzorno, Elizabeth A. Capaldi, David Rovnyak

**Affiliations:** 1 Department of Chemistry, Bucknell University, 1 Dent Drive, Lewisburg, PA 17837, USA; jcm065@bucknell.edu (J.C.M.); rag034@bucknell.edu (R.A.G.); sgd011@bucknell.edu (S.G.D.); plm015@bucknell.edu (P.L.M.); 2 Department of Biology, Bucknell University, 1 Dent Drive, Lewisburg, PA 17837, USA; jwr023@bucknell.edu (J.R.); pizzorno@bucknell.edu (M.C.P.); ecapaldi@bucknell.edu (E.A.C.); 3 Bruker Biospin, 15 Fortune Drive, Billerica, MA 01821, USA; Amy.Freund@bruker.com; 4 Program in Animal Behavior, Bucknell University, 1 Dent Drive, Lewisburg, PA 17837, USA

**Keywords:** European honeybee, *Apis mellifera*, NMR metabolomics, proline, extraction, entometabolomics, low-field NMR

## Abstract

The feasibility of metabolomic ^1^H NMR spectroscopy is demonstrated for its potential to help unravel the complex factors that are impacting honeybee health and behavior. Targeted and non-targeted ^1^H NMR metabolic profiles of liquid and tissue samples of organisms could provide information on the pathology of infections and on environmentally induced stresses. This work reports on establishing extraction methods for NMR metabolic characterization of *Apis mellifera*, the European honeybee, describes the currently assignable aqueous metabolome, and gives examples of diverse samples (brain, head, body, whole bee) and biologically meaningful metabolic variation (drone, forager, day old, deformed wing virus). Both high-field (600 MHz) and low-field (80 MHz) methods are applicable, and ^1^H NMR can observe a useful subset of the metabolome of single bees using accessible NMR instrumentation (600 MHz, inverse room temperature probe) in order to avoid pooling several bees. Metabolite levels and changes can be measured by NMR in the bee brain, where dysregulation of metabolic processes has been implicated in colony collapse. For a targeted study, the ability to recover 10-hydroxy-2-decenoic acid in mandibular glands is shown, as well as markers of interest in the bee brain such as GABA (4-aminobutyrate), proline, and arginine. The findings here support the growing use of ^1^H NMR more broadly in bees, native pollinators, and insects.

## 1. Introduction

With enormous losses of honeybees and native pollinators in North America, it is necessary to understand and respond to the causes of the decline in pollinator populations. Broadly, honeybee colonies weaken and die as a result of numerous stressors of various severity, including viral infection, pesticides, and invasive mites [1,2,3]. The complex interactions among these stressors and the causative tipping point that leads to hive collapse remain unclear. A confounding question is why, when many colonies face the same stressors, do some survive and others collapse? As this problem continues to engage researchers, more tools are needed to understand the relationships between hive stresses and bee health.

The study of aqueous metabolites of insects, which may involve whole insects or specific fluids, has been developing through the use of both mass spectrometry and NMR-based methods [4,5,6,7,8,9,10,11,12,13,14]. Studies may include a focus on specific organs such as the brain [4,5,10,15], which is particularly of interest because metabolic dysregulation has been attributed to this organ, particularly owing to neonicotinoids and pesticides [16,17].

Metabolomics has also been used to study insects in relationship to organisms in their surrounding environments, including interactions with phytochemicals [14,18], allowing for a wider perspective on honeybee colony collapses [7,19]. Despite the recent emergence of honeybee metabolomics, diverse applications are surfacing, such as honeybee gut microbiota studies [20,21], foodomics associated with honey [22] and royal jelly [23], and agricultural insecticide/pesticide effects [9,11,14,23]. Broadly, the discovery of biomarkers through studies of the metabolome of insects such as honeybees invites potential for improvements in health monitoring and stressor identification, addressing the honeybee colony crisis at hand [24].

Sensitivity considerations have emphasized the need for either mass spectrometry approaches [4,5] or specialized hardware [12]. To improve sensitivity as well as to measure average properties, pooling of multiple bees in samples is also an option [8,9,10,11,14,25]. The work reported here builds on a recent result in detecting proline in the honeybee brain by NMR [15] to further develop the feasibility of single bee and single bee brain NMR of aqueous metabolites on relatively conventional NMR instrumentation, employing high field (14.1 T) and low field (1.88 T) NMR instruments. The broader hypothesis driving the development of single bee NMR methodology is that interrogating the metabolome of a single bee is an important goal to enable a better understanding of individual variation, while also enabling study designs based on means of hive characteristics.

This work demonstrates that metabolomic studies of whole honeybees, portions of honeybees, and single honeybee brains using NMR have the potential to assess colony health while ensuring that single bee metabolomes can be characterized as a part of research studies employing groups and related statistical clustering analyses. Leveraging the preferred extraction protocol (acetonitrile:water in a ratio of 2:1) and simple mechanical homogenization, we observe strong recovery of metabolites with reduced interference from spectral features of sugars. The sensitivity of NMR allows for precise quantification and identification of 30-35 metabolites in whole forager bees and 20-25 for forager bee brains (Table S1 Supplementary Materials). We show representative examples of metabolomic ^1^H NMR data in honeybees, such as for sex-specific differences, stages of life cycle, and a viral biomarker in the bee brain. In light of continuing *A. mellifera* colony losses nationally, tools such as the metabolomic NMR analyses described here could be pivotal in uncovering pathologically relevant causes and complications of the population decline. We also highlight that benchtop NMR can be an accessible tool for broadening access to potent NMR metabolic information on bees, particularly for assessing a hive based on sampling a few individual bees and comparing to non-targeted reference data.

## 2. Results and Discussion

**Figure 1:**
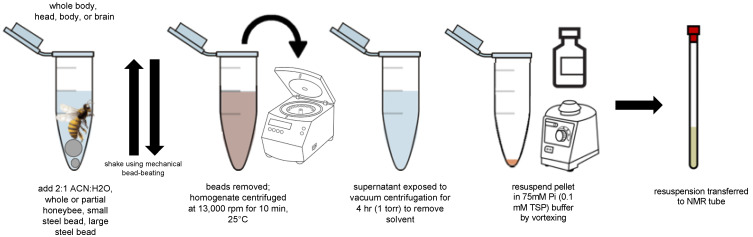
Organic extraction of whole bees as well as components (body, head, brain) was developed with acetonitrile:water mixtures. Prior work employed chloroform/methanol protocols, but acetonitrile achieves improved metabolite recovery and moderate reduction in sugars.

A set of NMR metabolomic applications is developed around the homogenization and extraction of aqueous metabolites of any of the following: (i) a whole bee, (ii) specific portions (e.g., body or head), or (iii) specific organs, e.g., the brain (Figure 1).

### 2.1. Extraction Methodology

Two methodologies for extracting aqueous metabolites from honeybee samples were considered, specifically (i) chloroform:methanol [26] extraction and (ii) acetonitrile extraction. Initial work with chloroform:methanol extraction [15] established a proof-of-principle for observing meaningful metabolites in homogenized bee samples. The chloroform:methanol method, however, involves significant sample handling and lower metabolite recovery. Approaches involving extractions with other solvents have been proposed for greater recovery of metabolites [27,28,29]. This work focused particularly on developing optimal acetonitrile extractions of individual bee samples. While the focus of this work was primarily honeybees (*Apis mellifera*), the spectra here support that ^1^H NMR of extracted single insects should be useful in broader insect metabolomics. Results from both extraction methods are considered in this work.

**Figure 2:**
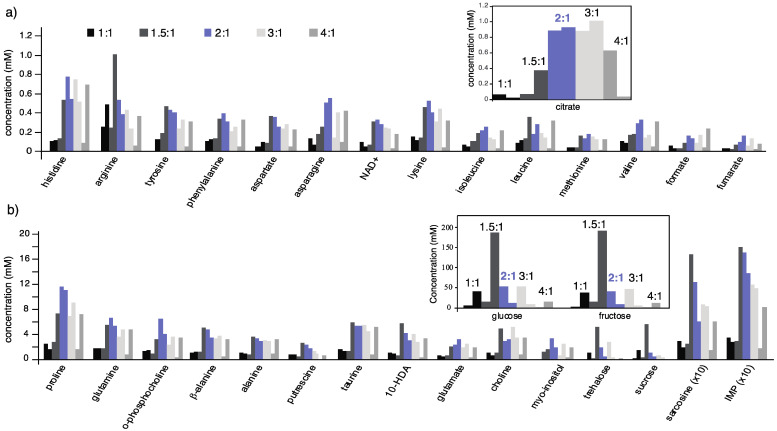
Concentrations of 32 metabolites obtained from the profiling of NMR spectra (pr-NOESY, 600 MHz, profiled with Chenomx 8.1 (Chenomx Inc., Edmonton, AB, Canada)) after the homogenization and extraction of single honeybees using different conditions. Data are shown for two bees in each of the following acetonitrile:water ratios: 1:1 (black), 1.5:1 (dark grey), 2:1 (blue), 3:1 (off-white), and 4:1 (light grey). Metabolites were grouped by relative concentrations: (**a**) relatively low concentrations, where the inset illustrates the data for a representative metabolite, and (**b**) intermediate concentrations, where the inset shows highly concentrated sugars. Note that the 2:1 ratio also decreased sucrose and trehalose in (**b**). The best overall recovery of metabolites was observed using the 2:1 acetonitrile to water ratio, although results with the 3:1 ratio were reasonably comparable. Spectra were acquired with 128 transients, 8 steady state scans, 13 s total delay (4 s acquisition and 9 s recovery), 2 s presaturation, and 100 ms NOESY mixing time. All bees are pollen foragers collected at the same time from one hive.

The recovery of aqueous metabolites was achieved by applying the acetonitrile extraction method initially to whole bees, enabling the profiling of about 35 metabolites (Figure 2). For examples of the spectral quality and some of the assignable metabolites relating to the samples considered in Figure 2, see also Figure S1 and Table S1 (Supplementary Materials). The good metabolite recovery observed confirms that the 1D NMR experiments (600 MHz, room temperature inverse probe) are sensitive enough to detect useful metabolic information for single honeybee samples with enough dynamic range in the detected concentrations to support analysis of trends. Many unassigned peaks remain in the spectra, begging the necessity of further work to assign the unknown peaks to uncover missing information of the metabolome. Suppression of background protein levels is evident in the clean baselines observed in resulting spectra (Figure S1, Supplementary Materials). Advances in developing alternative standards for metabolomic NMR that avoid the use of DSS and TSP are showing promising results and may enable a wider array of extraction methods in insects as well [30].

The optimization of the ratio of acetonitrile to water for the extraction procedure was assessed based on the ability of the solution to both reduce sugar levels and recover metabolites. A 2:1 acetonitrile to water ratio demonstrated good reduction in sugar signal levels and consistently showed highest recovery of metabolites. In comparison, the 3:1 ratio also performed well under the above-mentioned criteria, but the 2:1 ratio exhibited generally better results in this work. Homogenization and extraction of whole bees is attractive to avoid challenging procedures to selectively remove honeybee hemolymph in reproducible ways, but it introduces the anticipated obstacle of suppressing sugars. Specifically, extraction of whole bees requires the inclusion of honey stomach components in the larger sample, which contains nectar and is therefore expected to be a key source of sugars in some samples. High sugar concentrations can obscure metabolic information from less concentrated species (e.g., amino acids, energy metabolites) [8]. In whole bee samples, glucose and fructose were the most abundant sugars profiled, and it was critical that the 2:1 ratio reduced their levels; the trend of lower sugar levels with the 2:1 ratio was also seen for sucrose and trehalose (Figure 2).

In Figure 2, an example metabolite, citrate, is highlighted in the inset to exemplify the difference in metabolite recoveries that were broadly observed for the majority of metabolites in this work. Significant improvement is clearly seen for the 2:1 and 3:1 ratios of acetonitrile to water. Furthermore, recovering citrate and 10-HDA (10-hydroxy-2-decenoic acid) together in whole bee samples may be important, since these are of interest in geographic classification of royal jelly samples [23].

**Figure 3:**
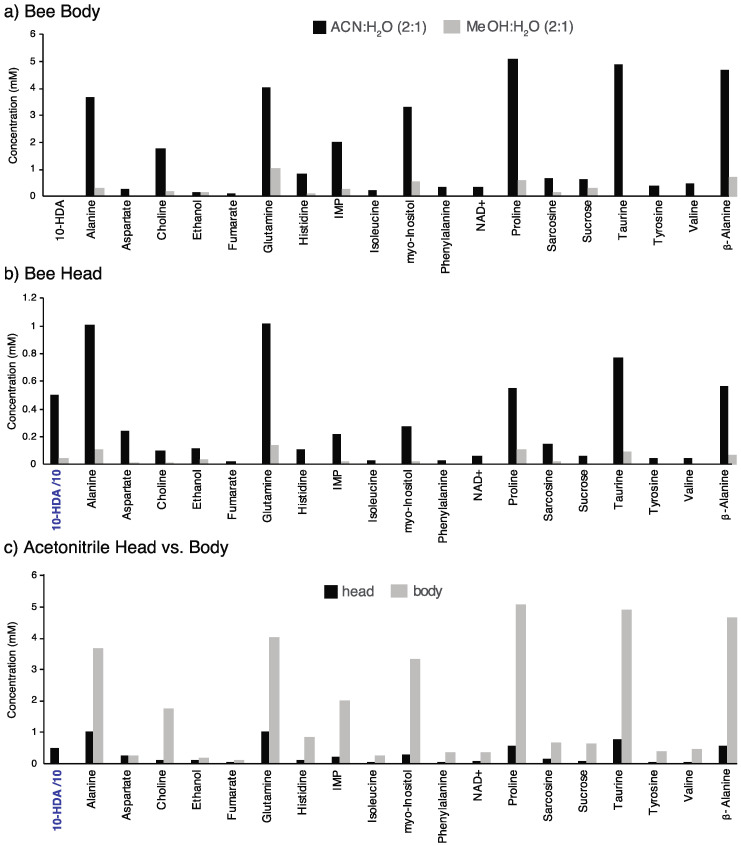
Representative metabolites recovered from head and body samples are shown. Results contrasting methanol:water (2:1) to acetonitrile:water (2:1) show better metabolite recovery from the acetonitrile treatment in both (**a**) bee bodies and (**b**) bee heads. In (**c**) the greater metabolite recovery in bodies is observed by comparing the acetonitrile results from (**a**) and (**b**). Spectra were acquired with 128 transients, 8 steady state scans, 13 s total delay (4 s acquisition and 9 s recovery), 2 s presaturation, and 100 ms NOESY mixing time. A notable difference is the detection of 10-HDA in the heads only. All bees are pollen foragers collected at the same time from one hive.

Representative profiling of metabolites (Figure 3) confirmed the ability to study the profile of a single honeybee body and a honeybee head separately. Methanol (2:1 methanol:water) and acetonitrile (2:1 ACN:water) extractions were employed and the success of their recovery was compared. Methanol extraction showed a poorer recovery of metabolites for both the head and body as compared to acetonitrile. Seven metabolites were recovered by the acetonitrile extraction but not with the methanol extraction, namely phenylalanine, asparagine, methionine, NAD+, taurine, fumarate (putative), and valine. While there was less recovery of metabolites with the methanol protocol, the methanol-extracted bee heads and bodies delivered relatively clean spectra with flat baselines. The acetonitrile extraction recovered more sugars such as glucose and sucrose, and about the same amount of trehalose. Sugar reduction was better with the methanol:water extraction; however, the somewhat higher sugar levels for acetonitrile-extracted bees was not detrimental to the overall quality of spectra and still resulted in greater overall metabolite recovery. Overall, it was a beneficial tradeoff to accept higher sugar levels in order to achieve better recoveries for all other metabolites using acetonitrile extraction.

The acetonitrile extraction showed good recovery of aqueous metabolites for the body and the head. As expected, based on the difference in volume of biological material, there were higher concentrations of metabolites observed in the body. Yet, the sensitivity of 600 MHz NMR was favorable for quantifying the metabolites found in the head, supporting the hypothesis that bee heads may be good candidates to be surrogates of dissected bee brains. As discussed in the next section, bee brains require time and skill to dissect. In contrast, the head can be separated reproducibly and simply from the bee body, and analyzing the head could be an attractive solution to improve the characterization of the bee brain. Overall, about 35 targeted metabolites were observed from the bodies and the heads. One notable difference was that 10-hydroxy-2-decenoic acid (10-HDA) was present in the head and not the body. This medium-chain fatty acid is a component of royal jelly, which is manufactured in the mandibular glands in the heads of worker bees [31]. So it is not surprising that we only detected 10-HDA in the forager heads. Royal jelly is fed to female bee larvae and sets in motion nutritionally dependent factors that lead to the development of queen bees. It is also given to adult queens throughout their lives. Prior studies employing mass spectrometry showed the presence of oxygenated fatty acids with six to twelve carbon atoms in the mandibular gland as well, which are fatty acids related to the royal pheromone [32]. Pesticides alter royal jelly, including decreased levels of 10-HDA [33], and the bee head NMR methods developed here could provide a new tool to study the effect of pesticides on royal jelly.

### 2.2. Honeybee Brain

Localizing metabolomic changes to the bee brain can test important hypotheses such as how viral infection changes expression patterns in the brain itself, but it raises the question of whether there is sufficient sensitivity to detect a meaningful subset of metabolites from a single bee brain by NMR methods. Note that here the dissection media for brains incorporates sugars [34], which could represent an exogenous source of additional sugars.

**Figure 4:**
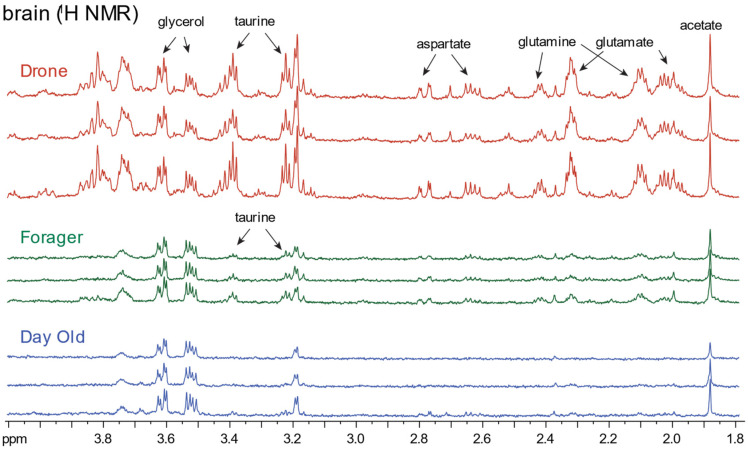
Representative ^1^H-NMR spectra (PURGE, 600 MHz, 14.1 T, 25 °C, 128 scans) of the aqueous phases of chloroform:methanol extractions from homogenized bee brain samples. Spectra show differences between day-old bee (worker), foragers (worker), and drones (males), particularly with respect to number of metabolites corresponding directly with brain size of bees. This series was conducted without a chemical shift standard in order to observe spectra in the absence of confounding signals/standards. Chemical shifts were referenced to a secondary standard.

Initial ^1^H-NMR work on dissected bee brains, extracted with the chloroform:methanol system (see Methods), recovered ca. 20 metabolites and discovered that proline was strongly upregulated in the brains of deformed wing virus (DWV)-infected bees [15] but did require spectra collected over approximately 2 h. Further examples of ^1^H-NMR spectra (600 MHz) of bee brains using chloroform/methanol extraction are shown in Figure 4, which contrasts developmental stages and sex of bees, where the clearest trend is the overall recovery of metabolites from progressively larger brain tissue samples that can be obtained. Sex-specific trends are supported but are outside the scope of this survey.

**Figure 5:**
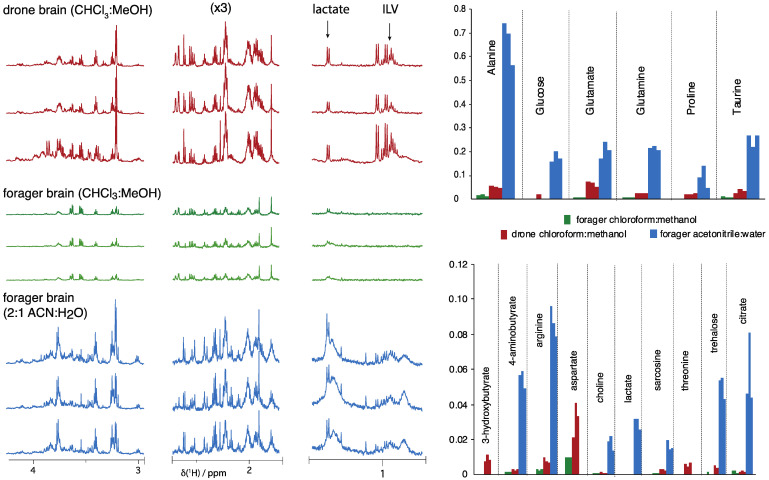
^1^H-NMR spectra (600 MHz, pr-NOESY, RT inverse probe) of aqueous metabolites extracted from dissected bee brains using both chloroform:methanol and acetonitrile:H_2_O (2:1) in both drones and foragers. For comparison, all spectra employed 128 scans and dissections preserved optic material (i.e., eyes). Note overall higher recovery of metabolites in the ACN:water extracts, where several amino acids (particularly ILV) are observed only in the ACN:water extracts.

The example spectra in Figure 4, which resulted from samples obtained by chloroform:methanol extraction, are clearly sensitivity-challenged and could limit future studies. Given the performance of the acetonitrile extraction illustrated in Figure 2 and Figure 3, the chloroform:methanol and acetonitrile extractions were compared for analysis of dissected bee brains (Figure 5). The forager brains extracted with ACN:H_2_O show higher overall recovery of metabolites. There is, in particular, a greater recovery of many amino acids with the acetonitrile extraction (Figure 5).

The remarkable metabolite levels recovered in the much larger drone brains, even using the chloroform/methanol extraction, can be seen in Figure 5. The bodies and brains of drones are considerably larger than those of workers and represent a greater overall sample. The chloroform:methanol method, while much less efficient than the acetonitrile method, may be preferred for concentrated samples such as drone brains. For example, notice in the spectra of acetonitrile extracts of foragers that broader signal regions ca. 1 ppm occur, which likely represent lipids and some residual proteins, but which are suppressed in the spectra of chloroform:methanol extracts. Since extracted drone brains have such high metabolite levels, it may not be necessary to resort to acetonitrile extractions, although acetonitrile extracts can approach ground truth (original metabolites levels) in sera and mock sera [27,29].

For forager brains, accepting higher sugar levels and some background signals with the acetonitrile method is a beneficial choice to obtain higher overall metabolite recoveries and overcome the sensitivity limitations noted in Figure 4. In fact, up to about 30 metabolites can be profiled under these conditions (600 MHz, inverse RT probe) in acetonitrile extracts of forager bee brains (Table S1 Supplementary Materials), where several are shown in the bar charts in Figure 5.

Reproducible excision of the bee brain must be conducted by an expert and may still yield samples that show variation in the recovered brain material. On average, a brain excision requires approximately 10–15 min. Commonly, such a dissection presents the opportunity to include or exclude the visual pigments of the eyes, which results in substantial changes in the spectra, notably, an overall increase in the signal intensities of all metabolites (Figure S2, Supplementary Materials). Potentially, some weak new signals are also observed when retaining the eyes, and we recommend that brain dissections retain the eyes as a strategy to recover as much brain matter as possible (Figure S2). Overall, the spectra in Figure 5 support that studies probing the sensitivity of the drone and forager brains to distinct hive states is feasible.

**Figure 6:**
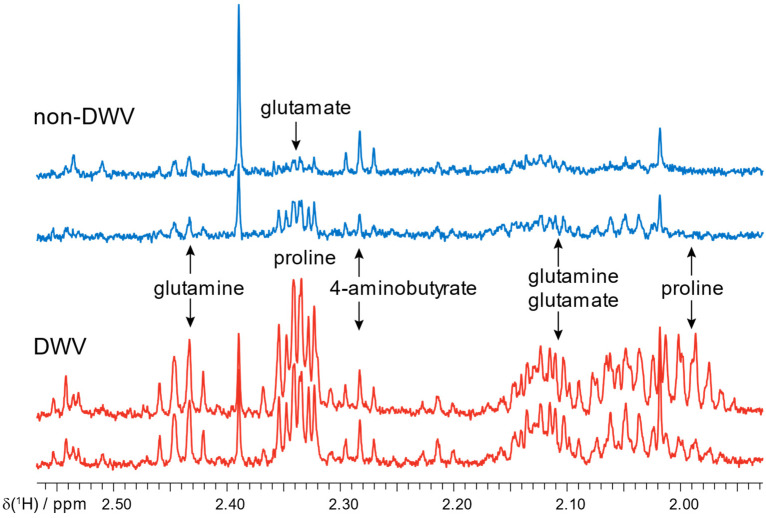
Representative spectra of extracted metabolites of forager brains with and without DWV show a particularly strong difference in the amount of proline, [15] although glutamine and glutamate are weakly suggested. In contrast, changes in 4-aminobutyrate (GABA), an important neurotransmitter, were not supported.

Emerging work is showing that bee brain metabolomics can report on distinct states including aggression [4] and viral infection [15]. Employing the earlier chloroform:methanol protocol to extract dissected forager brains, proline was observed to vary strongly between bees injected with deformed wing virus versus control bees, which correlated with increased expression of proline-rich peptides [15]. Representative spectra are illustrated in Figure 6. The ability of ^1^H NMR metabolomic methods to detect and localize a marker of DWV infection to the bee brain is an encouraging sign of the potential of this methodology to address important questions in bee health.

The fatty acid 10-HDA, a major component within secretions from worker bee mandibular glands, was not observed in brain dissections. In principle, incomplete removal of glandular materials from the brain tissues could result in finding 10-HDA in those samples. Note that brain glutamine and glutamate may be elevated as well with DWV infection, but additional work is required.

Among the metabolites detected in the brain NMR spectra in Figure 5 and Figure 6, the neurotransmitter 4-aminobutyric acid (also known as GABA) is quantified. GABA receptors are targeted by pesticides, and a disruption of GABA signaling leads to disruptions of motor functions in bees [35]. More broadly, it is widely recognized that NMR can offer complementary information to mass spectrometry methods in metabolomics [36]. In relation to this work, brain arginine is connected with the proboscis extension response [5], and this work indicates that brain arginine can be measured by NMR methods as well.

### 2.3. Low Field NMR

**Figure 7:**
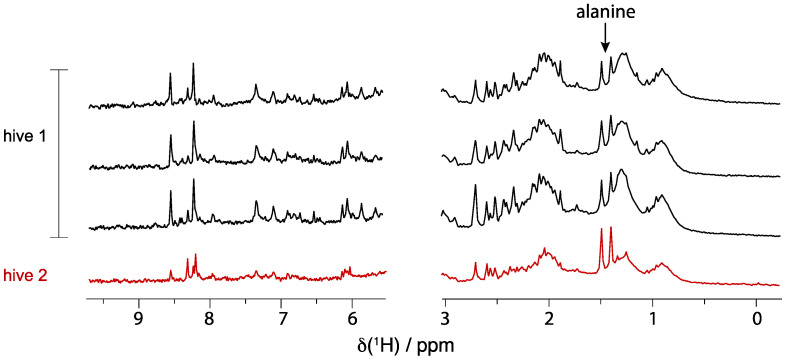
Representative low-field ^1^H-NMR spectra (80 MHz; composite pulse presaturation experiment with 512 transients, 2.5 s acquisition time, and 10 s combined saturation time and recycle delay) of the aqueous metabolome of acetonitrile extracts of single bees show good sensitivity in short acquisition times, accomplished in part by restricted volume sample tubes (5 mm/2.5 mm) employing 180 μL of sample.

Low field NMR spectroscopy offers fast, low-cost, sensitive NMR analysis and finds diverse applications [37,38]. We sought to determine the potential to conduct whole bee metabolomics at 80 MHz. To improve sensitivity, the dried extract was resuspended in 180 μL of buffer (commercially available phosphate buffer, pH 7.0, 10% D_2_O). Representative spectra of extracts of whole bees, sampled from two hives, employing 2:1 acetonitrile/water are illustrated in Figure 7.

The low field spectra in Figure 7 confirm that strong signals are detected and are sufficient to measure the state of a single bee. Some challenges are immediately recognized such as a strong influence of sugars in creating broad features in significant portions of the spectrum. Additionally, the need to work with highly concentrated samples may affect spectral quality (e.g., line width). However, the spectra also clearly show useful complexity throughout the spectral window that indicates the potential for low-field NMR to be a tool for non-targeted analyses. As a coarse proof-of-principle comparison, spectra of three bees from one hive are reasonably conserved compared to the spectrum of a bee from a distinct hive. It was unexpected to see the ability to detect alanine in these samples, showing an unanticipated potential for limited targeted study as well by such instrumentation and methods.

### 2.4. Other Bees and Pollinators: Carpenter Bee

**Figure 8:**
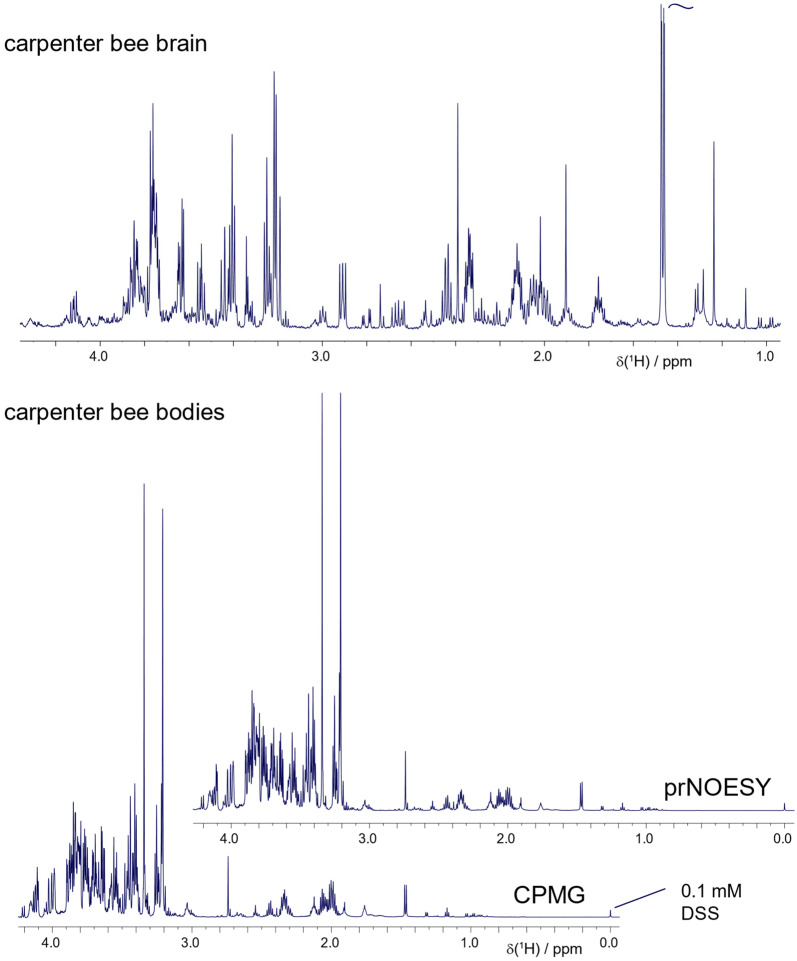
Larger bees, notably the carpenter bee, are particularly amenable to extraction and NMR analysis, where rich metabolite recovery was obtained from both a body and a brain. The typical 0.1 mM DSS standard concentration may not be optimal. Here, the chloroform:methanol approach was employed. The CPMG method results in highly similar spectra to pr-NOESY, supporting that the chloroform:methanol extract, although inefficient, has effectively suppressed proteins and lipids.

The application of such methodology to other bees such as the common carpenter bee, *Xylocopa virginica*, has been explored for feasibility using the chloroform:methanol method. The brain is even more accessible by dissection in carpenter bees due to their size and can produce remarkable spectra (Figure 8), while the metabolite recovery from just the carpenter bee body alone significantly exceeded the 0.1 mM DSS standard, which was well-suited for the honey bee studies in this work. As was the case for drone brain extracts in Figure 5, the chloroform:methanol extraction method may offer advantages in studying very rich samples such as the carpenter bee, due to its stronger reduction in sugar levels and background lipids and proteins.

## 3. Materials and Methods

### 3.1. Homogenization

A home-built, linear-oscillating bead-beating apparatus was developed for homogenization of single bees in typical 1.5 mL microcentrifuge tubes and employed stainless steel beads of varying diameters (⅛ and 3/16″).

### 3.2. Chloroform/Methanol Extraction

In initial work, a modified protocol by Bligh and Dyer [26] was used, employing a chloroform:methanol solution in a 1:2 *v*/*v* ratio (solution A). This protocol is described elsewhere [15] but is summarized here for completeness. First, 0.5 mL of solution A and two small steel beads were added to a single bee brain in a microcentrifuge tube. The sample was homogenized for 60 s and then 0.5 mL of chloroform was added, followed by vortex agitation. Then, 0.5 mL of dd-H_2_O was added, followed by vortex agitation and centrifugation at 4 °C (2000 RCF) for 2 min. Authentic lower phase, a cleaning solution that will be referred to as solution B, was prepared by repeating the steps above in a separation funnel using the pure solvents and isolating the lower phase. The upper layer of the homogenized bee brain sample following centrifugation was transferred to a new microcentrifuge tube, to which 0.5 mL of solution B was added and the above steps repeated to further wash and purify the aqueous metabolites. Following the wash step, the upper layer was transferred to a fresh tube and subjected to vacuum centrifugation for 4 h to remove solvent. For bee bodies, one large (diameter = ⅛″) and one small (diameter = 1/16″) steel bead were used for homogenization, which was extended to 3 min, with all other steps above being the same.

### 3.3. Acetonitrile Extraction

Optimization of an organic extraction using acetonitrile (HPLC-grade) and water yielded a reproducible method for preparing whole bees, bee heads, bee bodies, and bee brains for NMR experiments. An acetonitrile:water solution was optimized to a ratio of 2:1 *v*/*v* (solution C). To a microcentrifuge tube with biological bee material, 1.2 mL of solution C was added along with one large and one small steel bead. The bee material was homogenized using the mechanical bead-beater first for 30 s and then two additional times for 20 s each. The beads were removed, and the homogenized material was centrifuged for 10 min at 13,000 rpm on a benchtop centrifuge (~18,000 rcf). Additional spins were occasionally employed if the pellet was not well formed. The supernatant was exposed to vacuum centrifugation (4 h, 1 torr, 25 °C) and stored at −80 °C. Pellets were resuspended in 600 μL with TSP buffer (99% D_2_0, 0.1 mM TSP(IUPAC: sodium 3-trimethylsilylpropionate; also abbreviated as TMSP), 75 mM phosphate, pH 7.4). Vortex agitation aided in resuspension (~1 min). The resuspension was centrifuged for 2–5 min at 10,000 rpm (10,600 rcf) on a benchtop centrifuge to reduce particulates prior to transferring to 5 mm NMR tubes.

### 3.4. NMR Spectroscopy

Samples were resuspended in 0.5 mL of NMR buffer (99% D_2_O, 0.1 mM DSS (IUPAC: sodium 3-(trimethylsilyl)propane-1-sulfonate; also known as sodium 2,2-dimethyl-2-silapentane-5-sulfonate), 75 mM phosphate, pH 7.4) and transferred to glass NMR tubes. In some cases, the NMR buffer employed 100 mM phosphate. One-dimensional presaturation-NOESY ^1^H NMR spectra were acquired on a 14.1 T spectrometer (600 MHz for ^1^H, Varian Inc., Palo Alto, CA, USA, DDR1/VNMRS generation console, vnmrj 3.2) using an inverse probe. Unless otherwise noted, brain spectra generally utilized 512 transients (chloroform:methanol extract) or 128 transients (acetonitrile extract) and 32 steady-state transients, 4 s receiver time, a saturation period of 2 s, a NOESY mixing time of 160 μs, and a recycling delay of 9 s. Spectra were profiled manually by JCM, RAG, SGD, and DSR using the Chenomx NMR Suite 8.1 (Chenomx, Edmonton, AB, Canada) and are reported as concentrations (sample volume 0.5 mL). Analysis employed line broadening (0.5 Hz), phasing, baseline correction (spline), and reference deconvolution of the 0 ppm peak. Profiling is restricted to unambiguously assignable metabolites, e.g., unique and multiple signal clusters, and a limited number of putative singlets (see Table S1 Supplementary Materials). Low-field NMR was conducted at 1.88 T (80 MHz), wherein samples were resuspended in 180 μL of NMR buffer (commercially made phosphate buffer, 10% D_2_O, pH 7.0) and transferred to a glass NMR microtube (5 mm/2.5 mm NMR tube). One-dimensional composite presaturation ^1^H NMR spectra were acquired on a F80 spectrometer with TopSpin 4.1.3 software (1.88 T; Bruker BioSpin Corp., Billerica, MA, USA,), and using a 5 mm double-resonance H/C probe. Spectra utilized 512 transients, 2.5 s receiver time, and 10 s combined saturation and recycling delay.

### 3.5. Brain Dissection

Honeybees were placed in individual vials and placed on ice for 20 min to immobilize them using cold anesthesia. Heads were separated from the bodies using iridectomy scissors. While floating in cold bee saline [34], the brains were dissected from the head capsules using forceps: glands, muscles, and other connective tissues were removed from the brain tissue. Dissections were performed by the following authors: E.A.C., S.G.D. and J.R.

### 3.6. Hive Management

Unless otherwise stated, honeybee workers (*Apis mellifera* L.) were considered for the studies herein. Bees in the United States originate from a mixed assemblage of European, or western honeybee subspecies, including those described as Italian, Carniolan, and Russian. The bee colonies in the Bucknell apiary were headed by hybrid queens (primarily Italian, based on behavioral observations) and were maintained using standard beekeeping techniques in 10-frame Langstroth colonies; however, the hives were not medicated against any diseases or parasites. Some parts of the study employed different hives in different years. Pollen foragers were identified by the presence of pollen on the legs of bees as they returned to the hive. The bees were sampled at the hive entrance using individual vials and placed immediately on ice for cold anaesthesia prior to brain dissection.

## 4. Conclusions

Studying a whole bee or major components (head, body, or brain) of a single bee by NMR methods is demonstrated here under the hypothesis of sampling the metabolic state of the bee. We have validated that ^1^H-NMR (600 MHz) on a room temperature inverse probe has enough sensitivity to profile a useful subset of the aqueous metabolome using extracts of individual honeybees (*Apis mellifera*) and individual isolated parts and organs of the bee (head, body, brain). The single-bee approach avoids the need for pooling multiple bees and enables better understanding of biochemical traits in individual bees as well as the design of group-based studies designed around metabolite means. Metabolomic sample preparation involving acetonitrile:water extraction in a ratio of 2:1 was shown to be preferable in providing excellent metabolite recovery while also reducing sugar levels. Focusing on ^1^H 1D-NMR spectra, the profiling of 30–35 metabolites from whole forager bees and 25–30 metabolites in forager bee brains was possible under the conditions of this work, with some unassigned peak clusters that require the attention of further studies. Some notable metabolites that can be measured by ^1^H NMR metabolomics include citrate [23] in whole bees, 10-HDA in bee heads (mandibular glands), GABA in the bee brain, and arginine [5] and proline [15] in the bee brain. Due to sensitivity limitations from sampling a single bee, multidimensional NMR (e.g., 2D-NMR) is not favorable, particularly in bee brains, but its use merits further development. Single bee NMR should prove beneficial to enable future work focusing on group classification with the broad goal of advancing the understanding of the biochemical mechanisms underlying colony states and the assessments of honeybee colonies. The methodology presented here is sensitive to meaningful biological trends in honeybees (e.g., involving sex, age, DWV) and can be extended to other native pollinators and bees (e.g., carpenter), and should be of utility in other insect studies.

## Data Availability

The data presented in this study are available on request from the corresponding author. The data are not publicly available due to ethical.

## Supplementary Materials

The following are available online at https://www.mdpi.com/article/10.3390/metabo11120864/s1, Figure S1. Representative spectra as a function of acetonitrile:water ratio, Figure S2: Comparing *1*H NMR spectra of bee brain samples excised with and without eyes. Table S1: Typical metabolites profiled in whole bee, body, head, and brain.
