# Supplementary material for: Methodology for Single Bee and Bee Brain 1H-NMR Metabolomics
Source: Metabolites. 2021 Dec 13;11(12):864. doi: 10.3390/metabo11120864 (PMC8704342; doi:10.3390/metabo11120864)
Supplement: Supplementary file 1 [file metabolites-11-00864-s001.zip › metabolites-1467159-supplementary.pdf]

Supplemental Information For:

Methodology for Single Bee and Bee Brain ^{1}H-NMR Metabolomics

Jayne C. McDevitt [1], Riju A. Gupta [1], Sydney G. Dickinson [1], Phillip L. Martin [1], Jean Rieuthavorn [2], Amy Freund [3], Marie C. Pizzorno [2], Elizabeth A. Capaldi [2,4] and David Rovnyak [1,*]

1 Department of Chemistry, Bucknell University, 1 Dent Drive, Lewisburg, PA 17837, USA; jcm065@bucknell.edu (J.C.M.); rag034@bucknell.edu (R.A.G.); sgd011@bucknell.edu (S.G.D.); plm015@bucknell.edu (P.L.M.)
2 Department of Biology, Bucknell University, 1 Dent Drive, Lewisburg, PA 17837, USA; jwr023@bucknell.edu (J.R.); pizzorno@bucknell.edu (M.C.P.); ecapaldi@bucknell.edu (E.A.C.)
3 Bruker Biospin, 15 Fortune Drive, Billerica, MA 01821, USA; Amy.Freund@bruker.com
4 Program in Animal Behavior, Bucknell University, 1 Dent Drive, Lewisburg, PA 17837, USA
* Correspondence: drovnyak@bucknell.edu

This supplemental information contains two figures and one table that relate to the main text:

Figure S1. Representative spectra as a function of acetonitrile:water ratio

Figure S2: Comparing ^{1}H NMR spectra of bee brain samples excised with and without eyes

Table S1: Typical metabolites profiled in whole bee, body, head, and brain.

Figure S1. Representative spectra as a function of acetonitrile:water ratio

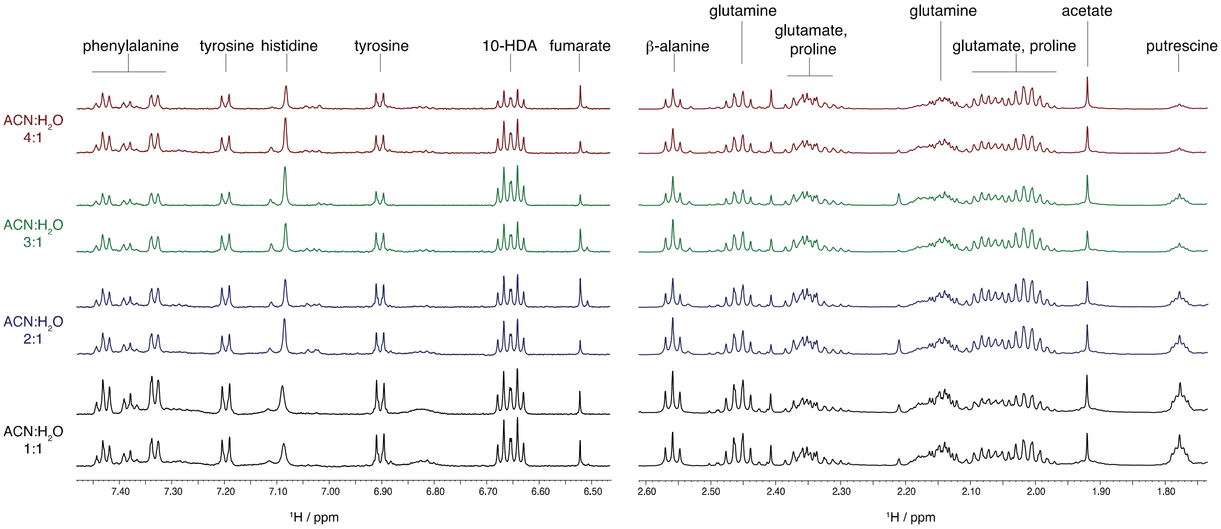

Figure S1. Representative spectra from samples analyzed in Figure 2 of the main text show good spectral quality that confirms good baseline properties, recovery of metabilites, and clearly assignable metabolites for subsequent profiling. Note visual inspection supports the preferred performance of the 2:1 ratio as many of the metabolites show decreased levels at higher ratios, particularly 4:1.

Figure S2: Comparing ^{1}H NMR spectra of bee brain samples excised with and without eyes

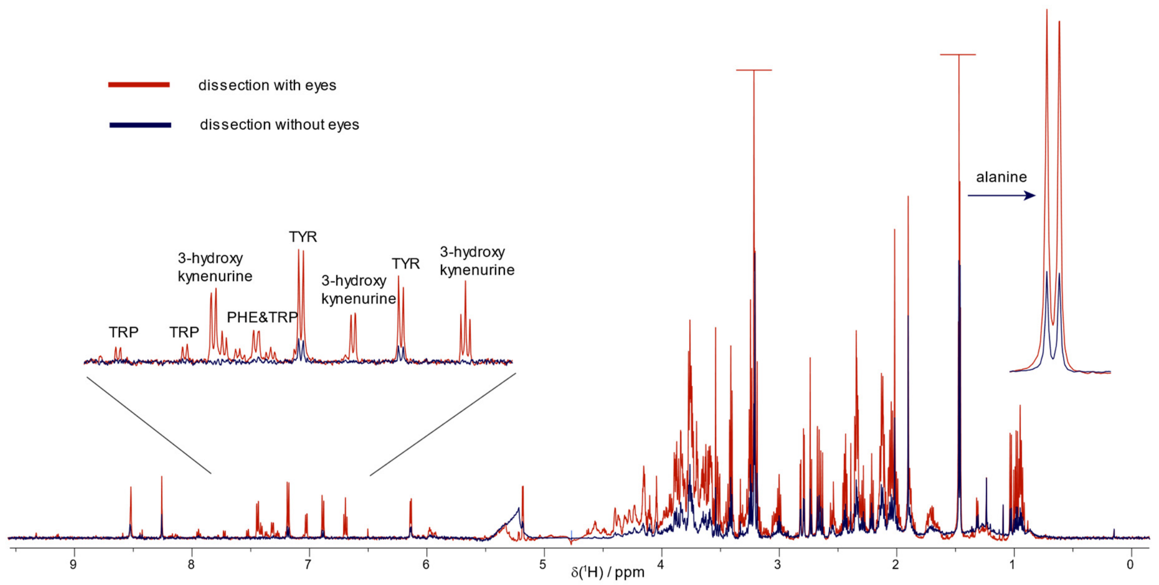

Figure S2. Dissecting the bee brain can involve including eyes or not. A comparison of ^{1}H NMR spectra from extracts (chloroform/methanol) of each case are shown, where the trend is that including eyes increases metabolite levels, due to recovery of overall more brain matter.

Table S1: Typical metabolites profiled in whole bee, body, head, and brain.

In the conditions of this study (single bee, 600 MHz NMR frequency, room temperature inverse probe), the routinely assignable and quantifiable metabolites (via Chenomx V8.1, Edmonton, Canada) are listed below, but the assignable metabolites are subject to variation in the bees and selected biological material.

Particularly in brain samples, the number of scans acquired and the amount of brain material recovered (i.e. **Figure S2**) can impact the observable metabolites.

Molecules typically assignable and profiled based on unambiguous matching of peak clusters (e.g. J couplings, multiple clusters) are listed in the table below. In a given bee, natural variation may lead to a small number occurring below detection limit.

**Whole bee, body, or head (2:1 acetonitrile:water extraction):***	**Notes:**
Energy, acids, other: acetate, 10-hydroxy-2-decenoic acid (10-HDA)[#], choline, citrate, formate, fumarate, IMP, myo-inositol, NAD+, o-phosphocholine, putrescine, sarcosine<br><br>*amino acids*: alanine, b-alanine, arginine, aspartate, asparagine, glutamate, glutamine, histidine, isoleucine, leucine, lysine, methionine, phenylalanine, proline, sarcosine, tyrosine, valine<br><br>*sugars*: fructose, glucose, sucrose, trehalose	[#]10-HDA originates from mandibular glands and is not detected in bodies.<br><br>[#]other medium chain dicarboxylic acids (suberate, pimelate, sebacate, azelate) are clearly supported in whole bee, body, and head samples but are not individually resolvable in these spectra.
**Brain (chloroform:methanol extraction):***<br><br>*Energy, acids, other*: acetate, choline, citrate, formate, fumarate, glycerol, IMP, lactate, o-phosphocholine, sarcosine, 3-hydroxykynenurine, 4-aminobutyrate<br><br>*amino acids*: alanine, b-alanine, arginine, aspartate, glutamate, glutamine, phenylalanine, proline, sarcosine<br><br>*sugars*: fructose, trehalose	Assumes retaining as much of the retina/optic neuropils (i.e. eyes) in dissection as possible
**Brain (2:1 acetonitrile:water extraction):***<br><br>*Energy, acids, other*: choline, citrate, formate, fumarate, glycerol, IMP, lactate, o-phosphocholine, sarcosine, 3-hydroxykynenurine, 4-aminobutyrate<br><br>*amino acids*: alanine, b-alanine, arginine, glutamate, glutamine, isoleucine, leucine, lysine, phenylalanine, proline, sarcosine<br><br>*sugars*: fructose, trehalose	Assumes retaining as much of the retina/optic neuropils (i.e. eyes) in dissection as possible<br><br>Overall higher levels recovered in acetonitrile (**Figure 5** of main text).<br><br>Amino acids are improved relative to chloroform:methanol extraction, although aspartate was not detected using acetonitrile.
* acetate, formate, fumarate, pyruvate occur as singlets in isolated, well-conserved regions; additionally, putative glycine, succinate, and pyruvate are supported in these spectra.	
